# Lumbar Spinous Process Impaction Injuries Caused by Extension Stress in Adolescent Athletes: A Report of Two Cases

**DOI:** 10.7759/cureus.84363

**Published:** 2025-05-18

**Authors:** Yuya Fukuda, Kinshi Kato, Kenichi Otoshi, Takuya Nikaido, Kazuyuki Watanabe, Hiroshi Kobayashi, Yoshihiro Matsumoto

**Affiliations:** 1 Department of Orthopedic Surgery, School of Medicine, Fukushima Medical University, Fukushima, JPN; 2 Department of Sports Medicine, School of Medicine, Fukushima Medical University, Fukushima, JPN; 3 Department of Research for Spine and Spinal Surgery, School of Medicine, Fukushima Medical University, Fukushima, JPN

**Keywords:** adolescent, athlete, case report, lumbar spine, spinous process

## Abstract

Lumbar spinous process injuries in adolescent athletes are rare and primarily attributed to traction mechanisms during flexion stress. Here, we present two cases of spinous process contusion and fracture, believed to result from impaction of adjacent spinous processes during lumbar extension. Case 1 involved a 14-year-old male volleyball player who presented with localized tenderness between the L4 and L5 spinous processes and extension-induced lower back pain. Magnetic resonance imaging (MRI) revealed high signal intensity at the caudal aspect of the L4 spinous process on short-tau inversion recovery (STIR) sequences; however, computed tomography (CT) revealed no evidence of fracture. Symptoms fully resolved after three months of lumbar orthosis use combined with cessation of sports activities, as confirmed by follow-up clinical and imaging assessments. Case 2 involved an 11-year-old male basketball player who experienced acute lower back pain immediately after a hyperextension injury sustained during lifting in a hyperextended posture. MRI demonstrated high signal intensity at the cranial L4 spinous process on STIR sequences, and CT revealed a fracture line. Continued play with extension restriction using a lumbar orthosis resulted in bony union within three months. These cases highlight a novel mechanism involving impaction during lumbar extension for lumbar spinous process injury in adolescent athletes. Early recognition and conservative management focusing on extension restriction are key to achieving favorable outcomes.

## Introduction

The posterior elements of the lumbar spine play a key role in sagittal stability. During extension, spinous processes act as bony restraints by approximating and limiting hyperextension [1]. In flexion, the supraspinous and interspinous ligaments function as tension bands, resisting separation of the spinous processes and preventing hyperflexion [2]. Together, these structures maintain segmental alignment and protect against instability during dynamic sports movements. Isolated lumbar spinous process injuries are rare in children and usually result from high-energy trauma or flexion-induced avulsion [3-6]. Moreover, previous reports involving adolescent athletes have predominantly attributed such injuries to traction forces exerted by the supraspinous or interspinous ligaments during lumbar flexion.

To our knowledge, direct impaction of adjacent spinous processes during lumbar extension causing contusion or fracture without avulsion has not been previously reported in adolescents. Herein, we report two cases emphasizing the clinical features, imaging findings, management strategies, and significance of recognizing impaction-type injuries as a novel entity in young athletes.

## Case presentation

Case 1 

A 14-year-old right-handed male volleyball player, serving as a front-row offensive player, developed subacute lower back pain after increased spike training. The pain persisted for three weeks despite physiotherapy, prompting referral for suspected lumbar spondylolysis.

Past Medical History

The patient had no significant past medical history.

Physical Examination

**Figure 1:**
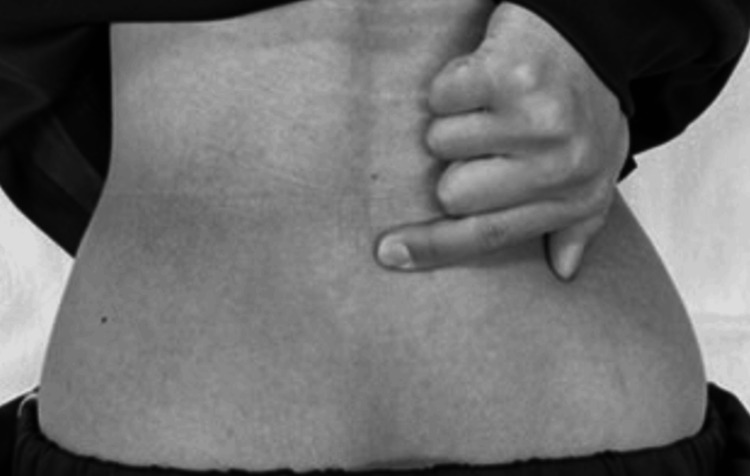
Physical examination findings in Case 1. The patient indicates localized tenderness between the L4 and L5 spinous processes.

Localized tenderness between the L4 and L5 spinous processes (Figure 1), on lumbar extension, and restricted right trunk rotation were observed. Flexion pain, Kemp’s sign, femoral nerve stretch test, and straight leg raise (SLR) test results were negative. However, SLRs were limited to 60° bilaterally due to hamstring tightness, with a heel-to-buttock distance measuring approximately 6 cm on both sides.

Imaging

**Figure 2:**
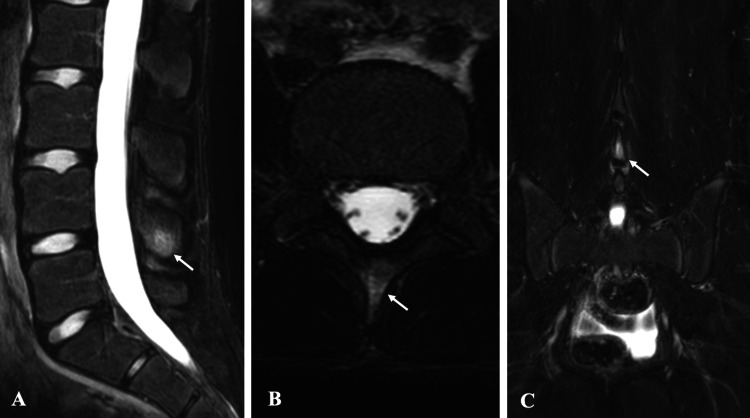
Pre-treatment MRI findings in Case 1. STIR sequence MRI reveals high signal intensity at the caudal aspect of the L4 spinous process: (A) sagittal view, (B) axial view, and (C) coronal view. MRI, magnetic resonance imaging; STIR, short-tau inversion recovery

**Figure 3:**
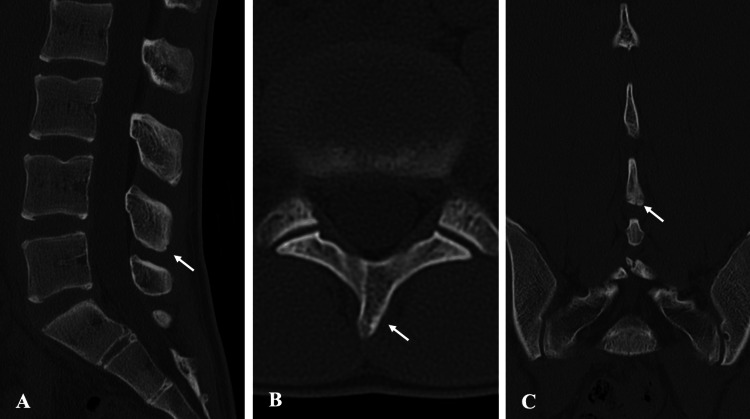
Pre-treatment CT findings in Case 1. CT shows no evidence of fracture at the corresponding site: (A) sagittal view, (B) axial view, and (C) coronal view. CT, computed tomography

Magnetic resonance imaging (MRI) revealed no signs of spondylolysis or disc bulging; however, high signal intensity was observed caudal to the L4 spinous process on short-tau inversion recovery (STIR) sequences (Figure 2). Computed tomography (CT) showed no fracture (Figure 3). Skeletal maturity corresponded to Risser stage 3.

Diagnosis

The patient was diagnosed with a contusion of the L4 spinous process.

Treatment and Outcome

**Figure 4:**
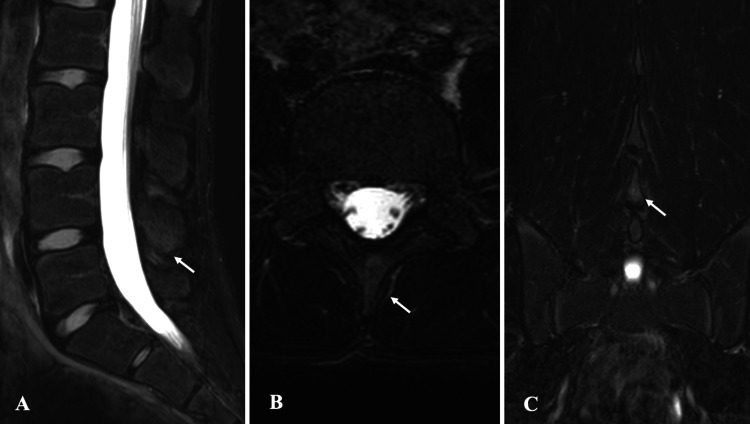
MRI findings in Case 1 three months after treatment. STIR MRI shows complete resolution of high signal intensity at the L4 spinous process: (A) sagittal view, (B) axial view, and (C) coronal view. MRI, magnetic resonance imaging; STIR, short-tau inversion recovery

Initially, the patient continued athletic activities; however, symptoms persisted for approximately three to four weeks. Therefore, he was referred to our department. Although the patient temporarily continued competing due to an upcoming event, substantial rest from sports began six weeks after symptom onset. Treatment included a soft lumbar orthosis with four aluminum stays (Maxbelt S3, SIGMAX, Tokyo, Japan) and physiotherapy. Physiotherapy focused on reducing extension stress on the lumbar spine by increasing thoracic, scapulothoracic, and rib cage mobility and improving flexibility of the iliopsoas and quadriceps. Additionally, anterior core strengthening exercises targeting the rectus abdominis and oblique muscles were incorporated to enhance anti-extension control and reduce the mechanical loading on the spinous processes.　Symptoms persisted up to eight weeks, and gradual improvement was observed thereafter. At 14 weeks after symptom onset, both clinical symptoms and MRI findings had completely resolved (Figure 4). The patient successfully returned to competitive sports without recurrence.

Case 2

An 11-year-old right-handed male basketball player developed acute lower back pain after lifting a heavy object while in a hyperextended posture. Symptoms persisted even after two weeks of rest.

Past Medical History

The patient had no significant past medical history.

Physical Examination

Localized tenderness between the L4 and L5 spinous processes and pain on lumbar extension were observed. Flexion pain, Kemp’s sign, femoral nerve stretch test, and SLR test results were negative. SLRs were limited to 80° bilaterally, and the heel-to-buttock distance was almost zero on both sides, indicating no tightness of the bilateral hamstrings and quadriceps femoris muscles.

Imaging

**Figure 5:**
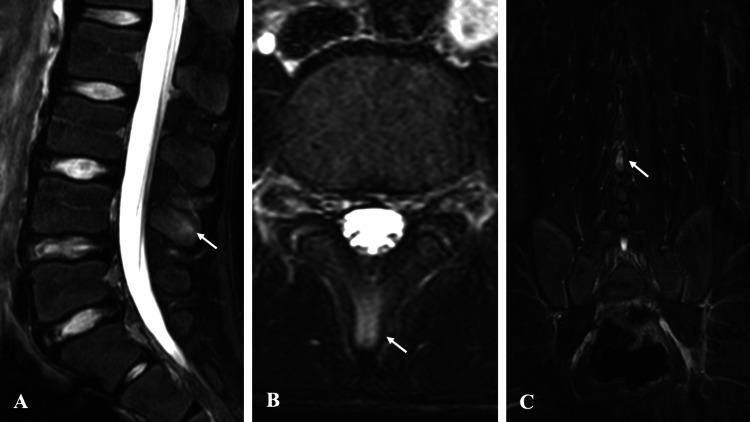
Pre-treatment MRI findings in Case 2. STIR sequence MRI reveals high signal intensity at the cranial aspect of the L4 spinous process: (A) sagittal view, (B) axial view, and (C) coronal view. MRI, magnetic resonance imaging; STIR, short-tau inversion recovery

**Figure 6:**
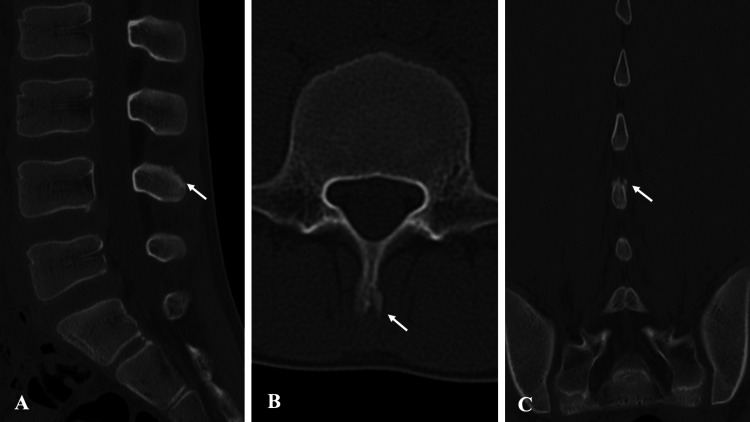
Pre-treatment CT findings in Case 2. CT shows a fracture line at the same location: (A) sagittal view, (B) axial view, and (C) coronal view. CT, computed tomography

High signal intensity was observed at the cranial aspect of the L4 spinous process on STIR sequence MRI (Figure 5), and CT revealed a fracture line at the same site (Figure 6). Skeletal maturity was assessed as Risser stage 0.

Diagnosis

The patient was diagnosed with a fracture of the L4 spinous process.

Treatment and Outcome

**Figure 7:**
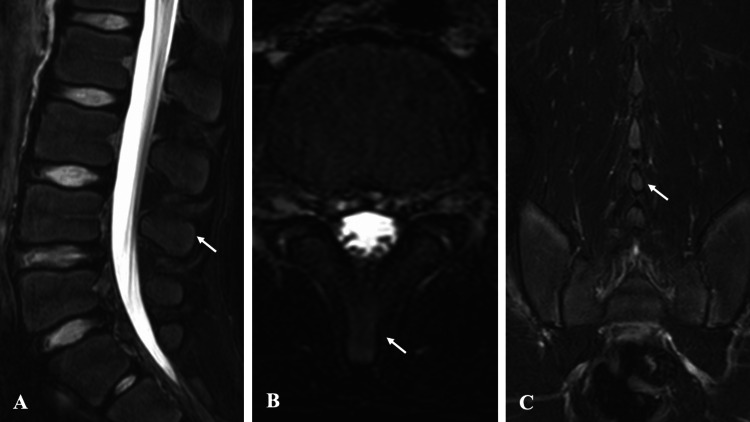
MRI findings in Case 2 three months after treatment. STIR sequence MRI shows resolution of high signal intensity at the L4 spinous process: (A) sagittal view, (B) axial view, and (C) coronal view. MRI, magnetic resonance imaging; STIR, short-tau inversion recovery

**Figure 8:**
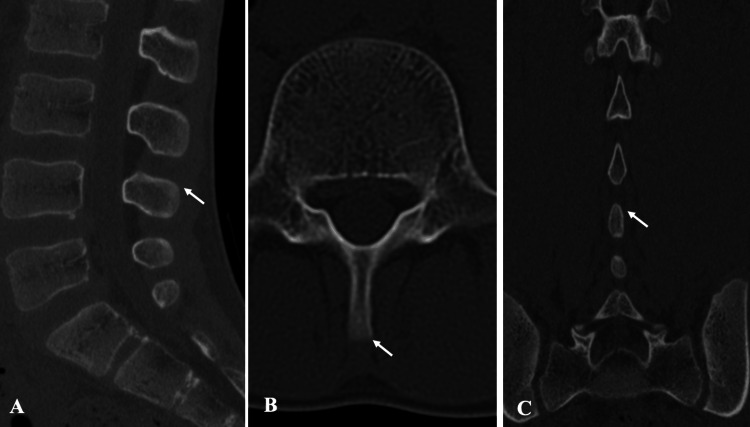
CT findings in Case 2 three months after treatment. CT confirms bony union at the fracture site: (A) sagittal view, (B) axial view, and (C) coronal view. CT, computed tomography

Even after symptom onset, the patient continued athletic activities for approximately two to three weeks before referral. Conservative treatment was initiated early using a soft lumbar orthosis with four aluminum stays (Maxbelt S3, SIGMAX) to restrict extension and physiotherapy, while allowing modified sports participation with restricted lumbar extension. Symptoms gradually improved, and both clinical and imaging abnormalities resolved at 14 weeks after symptom onset (Figures 7-8). The patient returned to full sports participation without recurrence.

## Discussion

Spinous process injuries in adolescent athletes can result from both flexion-induced traction and extension-induced direct impaction. Anatomical and mechanical factors, including a relatively large flexion-extension range of motion in the lower lumbar segments [7], shorter interspinous distances at the caudal levels, especially during extension [8], and increased lumbar lordosis during adolescence [9], may contribute to the occurrence of such injuries. These anatomical predispositions, combined with repetitive hyperextension movements inherent in sports such as volleyball (spiking) and basketball (jumping and rebounding), as well as training involving forced lumbar extension, may facilitate impaction between adjacent spinous processes and result in injury.

Clinical presentation and imaging findings are critical in differentiating impaction injuries from other causes of lower back pain. Differential diagnoses included lumbar spondylolysis, intervertebral disc herniation, facet joint syndrome, interspinous pain, and nonspecific mechanical lower back pain [10-12]. Lumbar spondylolysis and intervertebral disc herniation are typically diagnosed using MRI; however, no such findings were observed in either case. In contrast, facet joint syndrome, interspinous pain, and nonspecific mechanical low back pain generally lack specific imaging findings and are often diagnosed by exclusion based on clinical examination. Although spondylolysis is a common cause of extension- and rotation-induced low back pain in adolescent athletes [11], the presence of midline localized tenderness directly over the spinous processes, combined with extension-induced pain and corresponding MRI changes, favors a diagnosis of spinous process contusion or fracture. In particular, the absence of flexion pain helps differentiate these cases from traction-related injuries. Although plain radiographs may fail to detect subtle lesions, MRI is valuable for detecting bone marrow edema without cortical disruption, and CT is useful for confirming the presence of fracture lines. Although not used in our patients, ultrasound imaging is a potential adjunct for evaluating spinous process avulsion or subperiosteal lesions [13]. Moreover, functional imaging of the lumbar spine might have been useful for a more detailed assessment of the pathology; however, it was not performed in our patients. While Baastrup’s disease (*kissing spine syndrome*) in adults also involves spinous process contact during lumbar extension [14], it typically arises from chronic degenerative changes associated with aging. In contrast, the current cases represent acute or subacute, sports-related mechanical impaction injuries occurring in skeletally immature individuals without an underlying degenerative pathology or spinal malalignment. Repetitive mechanical stress and localized hematoma in such injuries may predispose to heterotopic ossification, a possibility warranting long-term follow-up [15].

Early diagnosis and appropriate mechanical unloading are essential for achieving favorable outcomes. Conservative measures, particularly lumbar extension restriction, can promote a complete recovery. Previous reports on clay-shoveler fractures in adolescents suggest that most cases respond favorably to conservative treatment [4,5,16]; however, delayed union occasionally necessitates surgical intervention [6]. Case 1 initially continued athletic activity after diagnosis but subsequently achieved symptom resolution following activity restriction and bracing. In contrast, Case 2 successfully continued modified sports participation while limiting lumbar extension through bracing. In addition to mechanical restriction, physiotherapy may be beneficial because tightness of the hip flexors is associated with increased lumbar lordosis in adolescents [17]. In Case 1, the tightness of the quadriceps was noted, which may have contributed to the development of impaction. The physiotherapy program included mobility exercises for the thoracic spine and shoulder girdle, stretching of the iliopsoas and quadriceps muscles to reduce anterior pelvic tilt, and anterior core strengthening (e.g., rectus abdominis and obliques) to enhance anti-extension control. These findings suggest that early diagnosis and appropriate conservative treatment, including physiotherapy and extension restriction, improve prognosis.

Interestingly, neither patient reported pain during lumbar flexion, despite the spinous processes being connected by ligaments that are placed under tension during such motion. We speculate that the injuries in these cases were primarily osseous, with minimal or no involvement of the supraspinous and interspinous ligaments, as supported by the lack of soft-tissue changes on MRI. Thus, extension-provoked compression, rather than flexion-related tension, appears to be the dominant pain mechanism. Although an extension-induced impaction mechanism appears to best explain the clinical and imaging findings in our cases, alternative or additional forces cannot be excluded. Traction stress at the supra- or interspinous ligament or combined flexion-extension loading could potentially contribute to micro-injuries during the spinous process, particularly in skeletally immature individuals. Further biomechanical analysis and advanced imaging may be necessary to clarify the precise pathomechanism.

## Conclusions

These cases reveal a novel injury mechanism in adolescent athletes: direct impaction of the adjacent lumbar spinous processes during extension. Three key findings were elucidated: first, spinous process injuries can result not only from flexion-induced traction stress but also from extension-induced impaction stress; second, clinical presentation and imaging findings are crucial for differential diagnosis; and third, early diagnosis followed by appropriate extension restriction can lead to favorable clinical outcomes.

In adolescent athletes with midline low back pain and extension-induced symptoms, lumbar spinous process impaction injury should be considered in the differential diagnosis. Recognizing this mechanism can enhance diagnostic accuracy and guide personalized treatment strategies for young athletes.

However, standardized criteria regarding the optimal duration of rest and immobilization, timing of return to sports, and long-term outcomes have yet to be established. Furthermore, the proposed impaction mechanism remains hypothetical because it is based on clinical and imaging correlations rather than biomechanical validation. Further confirmation through larger cohort studies and dynamic imaging analysis is necessary to clarify its generalizability and pathophysiological basis.
